# Does Functionality Appreciation Mediate the Relationship Between Breastfeeding Attitudes and Breastfeeding Intentions?

**DOI:** 10.3390/nu18081248

**Published:** 2026-04-15

**Authors:** Cristian Di Gesto, Marta Spinoni, Caterina Grano

**Affiliations:** Department of Psychology, Sapienza University of Rome, 00185 Rome, Italy; marta.spinoni@uniroma1.it (M.S.); caterina.grano@uniroma1.it (C.G.)

**Keywords:** breastfeeding attitudes, breastfeeding intentions, functionality appreciation, positive body image, postpartum women

## Abstract

Background: This study aimed to contribute to the growing empirical interest in the role of positive body image in the context of breastfeeding. Research Aim: We examined the association between positive attitudes toward breastfeeding and breastfeeding intentions among postpartum women and investigated the mediation of functionality appreciation. Method: A total of 305 women who had given birth within the past 1 to 3 months (M = 34.11 years) participated in the study. Women completed a questionnaire assessing breastfeeding attitudes, appreciation of breastfeeding functionality, breastfeeding intentions, previous breastfeeding experience, as well as Body Mass Index and sociodemographic and obstetric characteristics. A mediation model was used to examine direct and indirect associations between attitudes toward breastfeeding, breastfeeding intentions, and functionality appreciation. Results: Results showed significant associations between positive attitudes towards breastfeeding, breastfeeding intentions, and functionality appreciation. Positive attitudes toward breastfeeding were positively associated with breastfeeding intentions (β = 0.63, *p* < 0.001) and with functionality appreciation (β = 0.51, *p* < 0.001), with functionality appreciation accounting for a significant indirect association (β = 0.21, 95% CI [0.14, 0.29]). Finally, previous breastfeeding experience was positively associated with breastfeeding intentions (β = 0.15, *p* < 0.001). Conclusions: This study marks the initial attempt to examine the significance of functionality appreciation in postpartum women, highlighting potential associations between breastfeeding attitudes and breastfeeding intentions. These findings may offer preliminary insights for future research and for informing the development of targeted interventions, although further evidence from more diverse populations is needed.

## 1. Background

Breast milk is recognized as an optimal source of nourishment for infants [1] since it is correlated with an array of health outcomes, including reductions in gastrointestinal and respiratory infections and asthma episodes [1]. More specifically, exclusive breastfeeding has been largely shown to be a significant protective factor for obesity, e.g., [2,3]. Indeed, studies have shown that children who were exclusively breastfed for the first six months of life had a lower likelihood of obesity compared to those who were exclusively formula-fed and those who received a combination of breast milk and formula [4,5]. In a study conducted across 22 countries, children who were never breastfed or breastfed for a shorter period had higher chances of developing obesity over time compared to those who were exclusively breastfed for at least 6 months [6]. Furthermore, a meta-analysis demonstrated that extending breastfeeding beyond the sixth month was linked to a 4.0% reduction in the likelihood of developing obesity per month over time [7]. The protective effect of breastfeeding on obesity is not limited to childhood but extends throughout the adult years [8]. A recent review found that longer breastfeeding duration was associated with lower Body Mass Index (BMI) trajectories and obesity rates up to age eighteen years old [9].

The World Health Organization (WHO) [10] suggests exclusive breastfeeding up to the age of 6 months, followed by continued breastfeeding alongside suitable complementary foods until the child reaches 2 years of age or beyond. According to the Centers for Disease Control and Prevention (CDC) [1], most mothers (83.2%) acknowledge engaging in breastfeeding at some point. Nevertheless, only approximately 25% of them exclusively breastfeed up to 6 months of children’s age [1]. Hence, numerous infants are receiving partial exposure to breast milk, and probably not as extensive as recommended by medical guidelines. Although mothers may have positive attitudes toward breastfeeding, various factors may interfere with their translation into behavioral intentions [11,12].

The act of breastfeeding is a physical process that involves women’s bodies and may influence their perceptions and emotions regarding physical appearance. Indeed, breastfeeding may include breast enlargement, weight fluctuations, nipple soreness, fissures, and mastitis-related pain [13,14], as well as concerns related to social exposure (i.e., embarrassment to breastfeeding in public) [15].

Changes in the body related to breastfeeding may conflict with the unrealistic Western cultural standards for women’s body image, leading to increased body concerns and dissatisfaction [16,17]. The concept of the bounce-back culture describes the societal pressure on new mothers to swiftly recover their pre-pregnancy body shape and size after childbirth, driven by unrealistic standards perpetuated by the media and social norms [18]. These expectations can, for instance, have a negative impact on breastfeeding practices. Indeed, some studies showed that women with more body or weight concerns wean their infants sooner [19,20,21] and avoid or shorten the breastfeeding duration [18,22].

Producing milk to nourish one’s child can be both challenging and demanding, yet at the same time highly rewarding and fulfilling [23]. Positive perceptions of one’s body may represent an important factor in understanding breastfeeding experiences.

The aim of the present study was to examine the associations between attitudes toward breastfeeding, functionality appreciation, and breastfeeding intentions in postpartum women, and to test whether functionality appreciation is indirectly associated with the relationship between breastfeeding attitudes and intentions.

Functionality appreciation includes favorable opinions of the body, acceptance of imperfections, and respect for the novel needs and functions of one’s own body [24]. During pregnancy, women begin to concentrate on their own body’s capacities to protect, nourish, and support the growth of the fetus [25]; this emphasis on functional appreciation may persist in the postpartum period, fostering feelings of pride and accomplishment for what one’s body can do, including the appreciation for the breastfeeding practice [26].

Despite the growing body of research on breastfeeding determinants, relatively little attention has been paid to positive body image constructs in this context. In particular, functionality appreciation—a key dimension of positive body image—has not been extensively examined in relation to breastfeeding attitudes and intentions. By integrating this construct within a strength-based framework, the present study offers a novel perspective on the psychological processes underlying breastfeeding intentions in postpartum women.

The present study contributes to the existing literature in several ways. While many studies on breastfeeding and body image have concentrated on negative facets of body image, such as body dissatisfaction, and operated under the assumption that poor body image may hinder breastfeeding, this approach is distinct. Notably, the present study explores how a positive dimension of body image (i.e., functionality appreciation) may play a mediating role in the relationship between positive attitudes toward breastfeeding and its related intentions.

Only three quantitative studies have explored the relationship between functionality appreciation and breastfeeding [23,27,28]. Of these, only one took into account attitudes toward breastfeeding; however, it did not consider intentions [23]. Studying behavioral intentions is, nonetheless, essential as they are considered the primary determinant of behaviors [11].

Fern et al. [27] found that women who breastfeed hold more favorable views regarding body functionality and exhibit greater appreciation. Rosenbaum et al. [28] also found that women whose breastfeeding expectations were met showed a higher appreciation for the functionality of their bodies. Moreover, Gillen and colleagues [23] showed that women’s positive attitudes toward breastfeeding were associated with body functionality awareness and appreciation and with fewer maladaptive weight-control behaviors. Nonetheless, the mediating role of positive body image in the relationship between attitudes toward breastfeeding and breastfeeding intentions has not been previously tested.

Aligned with previous contributions, e.g., [29], we hypothesized that positive attitudes towards breastfeeding are directly associated with higher breastfeeding intentions (Hypothesis 1). Additionally, we predicted that functionality appreciation would mediate this relationship (Hypothesis 2). In particular, we propose that a positive evaluation of breastfeeding may be associated with greater appreciation for the capabilities of one’s body, specifically in breastfeeding one’s child, which, in turn, may be associated with increased breastfeeding intentions. This hypothesis of an indirect association between attitudes towards breastfeeding and breastfeeding intentions via functionality appreciation is in line with studies indicating that women who had a positive evaluation of breastfeeding are less inclined to engage in self-objectification (i.e., the tendency to view themselves primarily as objects) [23], given the positive regard for the body associated with perceived breastfeeding success. More favorable perceptions of one’s appearance during breastfeeding may be associated with higher intentions to engage in this behavior.

**Figure 1:**
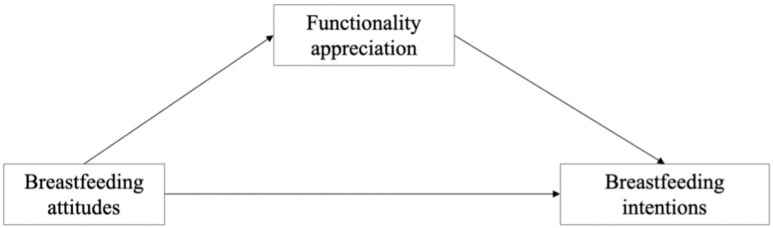
Conceptual model illustrating the hypothesized associations between attitudes toward breastfeeding (independent variable), functionality appreciation (mediator), and breastfeeding intentions (dependent variable), including both direct and indirect pathways. Previous breastfeeding experience was included as a covariate in the model.

Figure 1 depicts the hypothesized model, in which a direct and positive relationship between breastfeeding attitudes and breastfeeding intentions was hypothesized. Additionally, we predicted an indirect relationship mediated by body functionality appreciation.

## 2. Methods

### 2.1. Research Design

The present study was conducted using a cross-sectional design. The study adhered to the principles outlined in the Declaration of Helsinki, and the Institutional Review Board of the Department of Psychology, Sapienza University of Rome, granted approval for the study (Prot. n. 0002518, 16 November 2022).

### 2.2. Setting and Relevant Context

This study employed an online convenience sampling method. Convenience sampling encompasses various non-probability sampling techniques that are convenient for the researcher [30].

Postpartum women were recruited through various channels, including email, WhatsApp, Facebook, and other social media platforms. They were invited to participate in an online survey focusing on their childbirth experiences. All women were explicitly informed that their involvement was voluntary, their responses would remain anonymous, and confidentiality was guaranteed.

To be eligible for the study, participants had to be women aged 18 years or older, have given birth within the previous 1 to 3 months, and be able to speak and understand Italian fluently.

### 2.3. Sample

A group of 305 women who had given birth within the past 1 to 3 months was enlisted for the study. To be eligible for the study, participating mothers had to be at least 18 years old and be able to speak and understand Italian fluently.

The sample size was determined through a priori power analysis using G*Power (version 3.1.9.7). For a two-tailed bivariate correlation study with a medium effect size of 0.3, 138 participants would provide 95% statistical power, while a regression design with up to 10 predictors and a medium effect size of 0.15 would necessitate 172 participants. Therefore, the inclusion of 305 participants in the present study is deemed to provide adequate statistical power with confidence.

The sample was characterized by relatively high levels of education, which should be considered when interpreting the findings.

### 2.4. Measurement

BMI, Sociodemographic and Obstetric Characteristics. Height, weight, sociodemographic information (age, education level, marital status, nationality, place of residence), and childbirth-related information (parity and type of delivery) were collected. Education level was categorized into four groups: middle school, high school, undergraduate degree, and postgraduate education.

Breastfeeding attitudes. Attitudes toward breastfeeding over the ensuing six months were measured using nine semantic differentials (e.g., “useless/useful,” “unpleasant/pleasant”, “negative/positive”) based on previous breastfeeding studies [31,32]. Semantic differentials are widely used to assess evaluative attitudes through bipolar adjective pairs. Responses were averaged across items to obtain a mean score (α = 0.89; ω = 0.91).

Functionality appreciation. The Italian version [33] of the Functionality Appreciation Scale (FAS) [34] was used. The scale comprises 7 items (e.g., “I appreciate my body for what it is capable of doing”) rated on a 5-point scale (1 = strongly disagree; 5 = strongly agree) that measure the levels of an individual’s appreciation for what one’s body can do. Responses were averaged across items to obtain a mean score, with higher values indicating greater functionality appreciation (α = 0.89; ω = 0.91).

Mothers’ breastfeeding intentions. We used 3 items (e.g., “In the first 6 months of life, I intend to exclusively breastfeed my baby: answered on a 7-point Likert scale (1 = Definitely not; 7 = Definitely yes) based on previous studies [31,35]. The remaining items asked participants how strongly they wanted to exclusively breastfeed and how likely they thought it was that they would exclusively breastfeed. Responses were averaged across items to obtain a mean score, with higher values indicating stronger breastfeeding intentions (α = 0.90; ω = 0.94).

Previous breastfeeding experience. Two items based on previous research [36] were used to assess the frequency (e.g., “How frequently did you breastfeed?”) and quality (e.g., “If you have had breastfeeding experiences, how many of these would you consider positive?”) of breastfeeding past behavior. These two dimensions were multiplied to obtain a composite index reflecting both the extent and the perceived quality of prior breastfeeding behavior. This approach was adopted to capture a more ecologically valid representation of breastfeeding experience, as frequency alone may not fully reflect the subjective experience, and quality alone may not account for actual engagement in the behavior. The multiplicative combination allows for the identification of cases in which both frequent and positively experienced breastfeeding are present, distinguishing them from cases where only one of these aspects is high. For both items, responses were provided on a 6-point Likert scale (1 = none; 6 = many). The scores reported on the two items were multiplied. Higher scores on this scale indicate higher levels of previous breastfeeding experience (α = 0.86; ω = 0.88).

### 2.5. Data Collection

Women were required to provide a digital informed consent before accessing the formal questionnaire. This was facilitated through a provided web link that guided participants to the questionnaire administered through the Qualtrics@ platform [37]. The introductory page of the questionnaire detailed the overarching objectives of the research project, inclusion criteria, confidentiality safeguards, and researcher contact details. Individuals who confirmed that they were 18 years or older and expressed their consent were then directed to the subsequent page of the survey, which delved into inquiries regarding socio-demographic factors and details related to childbirth experiences. Subsequently, participants proceeded to complete the self-report questionnaires. No form of compensation was offered for participation.

The questionnaire took approximately 15 min to complete.

### 2.6. Statistical Analysis

The hypotheses were established prior to the data collection, and the analytical plan was predetermined. Descriptive statistics and zero-order correlations between the study variables were computed using IBM Statistical Package for the Social Sciences (SPSS), version 28.0 [38]. We tested the hypothesized conceptual model (mediation model) using the PROCESS macro for SPSS (Model 4) developed by Hayes [39], including previous breastfeeding experience as a covariate due to its well-established association with breastfeeding intentions. Other sociodemographic and obstetric variables (e.g., BMI, parity, type of delivery), although measured, were not included in the model in order to preserve model parsimony and avoid overfitting. Multicollinearity diagnostics were conducted to ensure the stability of the estimated parameters, and no issues were detected, with all variance inflation factor (VIF) values below the recommended threshold (i.e., <5) and tolerance values within acceptable ranges.

In our model, functionality appreciation was the mediator between attitudes toward breastfeeding (independent variable) and breastfeeding intentions (dependent variable).

Bias-corrected bootstrap confidence intervals (CIs) derived from 5000 bootstrap resamples were estimated to test for the significance of conditional direct and indirect effects. The effects are considered significant if the CI values do not include zero. Full mediation occurs when there is no direct effect present, whereas partial mediation involves the presence of an indirect effect along with a significant direct effect. The measure of the size of the mediation effect includes the mediated proportion, which signifies the fraction of the total effect that can be attributed to the indirect effect.

## 3. Results

### 3.1. Descriptive Statistics and Correlational Analyses

Table 1 shows the demographic characteristics of the sample. The average age of the sample was 34.11 (SD = 2.19) years, with an age range between 21 and 44.

The type of delivery was categorized as spontaneous vaginal delivery, induced vaginal delivery, elective cesarean section, and emergency cesarean section. Although the number of emergency cesarean sections was relatively small, this distinction was retained to reflect clinically meaningful differences in childbirth experiences.

Most participants had a high school or university-level education, with a substantial proportion holding postgraduate qualifications. The majority of the sample was married or cohabiting, and most women were primiparous. Regarding delivery mode, spontaneous vaginal delivery was the most frequent, followed by induced vaginal delivery and cesarean sections.

Descriptive statistics and Pearson’s correlations among the study variables are presented in Table 2. The findings showed that the Skewness and Kurtosis values were within acceptable ranges [40], indicating normal distributions of the scores for these variables. Significant and positive correlations were found between breastfeeding attitudes and both functionality appreciation and mothers’ breastfeeding intentions. Significant and positive associations were also found between functionality appreciation and breastfeeding intentions.

### 3.2. Mediation Model

Each relationship in the mediation model was statistically significant and consistent with the study hypotheses.

In line with Hypothesis 1, the total effect of breastfeeding attitudes on breastfeeding intentions was significant (c = 0.63, *p* < 0.001). When functionality appreciation was included in the model, the direct effect remained significant, although reduced (c’ = 0.42, *p* < 0.001), indicating partial mediation.

Breastfeeding attitudes were positively associated with functionality appreciation (a = 0.51, *p* < 0.001), which in turn was positively associated with breastfeeding intentions (b = 0.41, *p* < 0.001).

The indirect effect was significant (indirect effect = 0.21, 95% bias-corrected CI [0.14, 0.29]), supporting Hypothesis 2.

Previous breastfeeding experience was positively associated with breastfeeding intentions (β = 0.15, *p* < 0.001).

**Figure 2:**
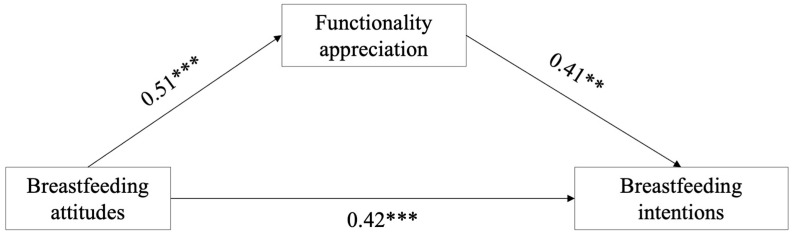
Mediation model examining the relationship between breastfeeding attitudes and breastfeeding intentions through functionality appreciation, controlling for previous breastfeeding experience. Standardized coefficients (β) are reported. ** *p* < 0.01, *** *p* < 0.001.

The model explained 49% of the variance in breastfeeding intentions. The results of the mediation model are shown in Figure 2.

Multicollinearity diagnostics indicated no issues among the study variables, with all VIF values below the recommended threshold.

## 4. Discussion

The current study represents the first attempt to assess the role of functionality appreciation in postpartum women, providing insight into potential associations through which breastfeeding attitudes may be associated with breastfeeding intentions and contributing to the growing empirical interest regarding the role of positive body image in the realm of breastfeeding experience [23,28]. However, these findings should be interpreted in light of the relatively homogeneous composition of the sample, which may limit their generalizability to populations with different socioeconomic and cultural backgrounds. Investigation of positive body image is crucial, as research is encouraged to move beyond a pathology-driven model, incorporating positive aspects into the exploration of human strengths [41,42]. Our findings showed positive associations among attitudes toward breastfeeding, breastfeeding intentions, and functionality appreciation. Furthermore, functionality appreciation statistically partially mediated the association between attitudes toward breastfeeding and breastfeeding intentions. This is particularly relevant given the recognized optimal nutritional benefits of breast milk for infants [1].

The observed direct association between positive attitudes toward breastfeeding and breastfeeding intentions (Hypothesis 1) is in line with previous research showing that these variables are closely related, e.g., [43,44]. Positive attitudes toward breastfeeding may be associated with greater awareness and understanding of the benefits of breastfeeding for both the mother and the infant. This finding is consistent with the possibility that knowledge about breastfeeding is associated with decision-making processes surrounding breastfeeding and may be associated with more favorable attitudes and stronger breastfeeding intentions [43,45].

Attitudes do not necessarily translate into actual intentions to breastfeed [46,47]. Indeed, other factors may shape the association between attitudes and intentions [48,49,50]. It is also important to acknowledge that breastfeeding intentions and behaviors are shaped not only by individual psychological factors, but also by broader structural and contextual influences, such as workplace policies, social norms, and access to support services.

Consistently, our findings indicated that functionality appreciation statistically partially mediated this association (Hypothesis 2). Women with more positive attitudes toward breastfeeding also reported greater appreciation of the functional and experiential dimensions of their bodies. In turn, greater functionality appreciation was associated with stronger breastfeeding intentions over the subsequent six months.

This finding is in line with Gillen et al. [23] and supports the notion that a positive evaluation of breastfeeding may be related to greater appreciation and respect for the novel needs and capabilities of postpartum bodies. During this crucial phase, a more positive disposition toward breastfeeding may be associated with greater attention to the physiological and practical dimensions of the body, and therefore with greater appreciation of the functional aspects of the breastfeeding experience [26]. Previous research suggests that breastfeeding may be associated with a sense of self-worth and satisfaction, acting as a testament to the effective functioning of women’s bodies [51]. A positive attitude, rooted in the recognition of the functional role of the female body in nourishing and sustaining an infant, may be associated with greater acknowledgment of body functionality during breastfeeding. In this vein, positive attitudes toward breastfeeding may co-occur with a greater acknowledgment of the functional aspects inherent in the postpartum period.

Regarding the link between functionality appreciation and breastfeeding intentions, a significant positive association has emerged. It is crucial to underscore that no studies have specifically investigated the relationship between functionality appreciation and intention to breastfeed in this particular context. Consequently, we are left to hypothesize explanations drawing from studies that have generally explored the role of functionality appreciation in postpartum women [23,28]. Higher levels of functionality appreciation have been reported to be associated with greater levels of body confidence [52]. In this view, higher functionality appreciation may be associated with greater confidence in one’s capability to breastfeed, which may in turn be associated with stronger breastfeeding intentions to engage in this behavior. When women feel more confident and comfortable with their bodies’ functionalities, they may report a more positive orientation toward breastfeeding and greater motivation to face possible challenges [23]. Moreover, greater appreciation for the innate functions of the body during breastfeeding may coexist with the rewarding physiological aspects involved (e.g., endorphin production, oxytocin), which may be associated with greater feelings of confidence and comfort in breastfeeding processes [53]. Finally, women who appreciate the functionality of their bodies while breastfeeding may also feel more connected to the biological and nurturing aspects of motherhood [28,54], which may be associated with greater commitment to breastfeeding.

Regarding the role of previous breastfeeding experience (in terms of frequency and quality of past behavior) as a covariate in the model, a positive association with breastfeeding intentions was found in line with previous studies [31,55,56]. Mothers who have had positive breastfeeding experiences in the past may be more inclined to plan to breastfeed in the future. Positive past experiences with breastfeeding may be associated with greater confidence and belief in one’s capacity to breastfeed, which may, in turn, relate to stronger breastfeeding intentions [57].

The relatively high correlations observed between previous breastfeeding experience and the main study variables (i.e., breastfeeding attitudes, functionality appreciation, and breastfeeding intentions) may reflect a meaningful conceptual overlap rather than a statistical artifact. Previous breastfeeding experience likely contributes to shaping both cognitive (attitudes) and embodied (functionality appreciation) representations of breastfeeding, which in turn are associated with behavioral intentions.

Importantly, multicollinearity diagnostics did not indicate any critical issues, with all variance inflation factor (VIF) values well below commonly accepted thresholds. This suggests that, although related, the variables capture distinct constructs and can be meaningfully included within the same model.

Nevertheless, the strength of these associations should be interpreted with caution, and future research may benefit from further disentangling the role of prior experience using longitudinal or experimental designs.

In interpreting these findings, it is important to distinguish between associations supported by the present data and broader theoretical implications.

The present study contributes to the literature by integrating a positive body image perspective—specifically functionality appreciation—into the investigation of breastfeeding attitudes and intentions in postpartum women. Rather than redefining established determinants of breastfeeding, our findings offer an incremental and theory-driven extension, highlighting functionality appreciation as a potential psychological correlate of the association between attitudes and behavioral intentions. In this sense, the study does not aim to replace existing explanatory models, but to complement them by introducing a strength-based perspective that has been largely overlooked in breastfeeding research. In particular, this study supports the idea that positive body image may represent a relevant correlate in breastfeeding-related attitudes and intentions. This focus on positive body image and functionality appreciation among postpartum women may have relevant implications for understanding maternal well-being and breastfeeding-related experiences. Positive body image is understood to consist of various components and is considered protective, especially in terms of health behaviors such as unhealthy eating habits [58]. Greater awareness and appreciation of body functionality during the postpartum period may be relevant for broader health-related attitudes and behaviors, although this possibility requires direct empirical examination. This is relevant, also considering the societal pressures and conflicting messages that women face from a young age regarding appearance and femininity, which can create a challenging environment that influences behaviors related to body image and health [18,59]. An appreciation for what the body is capable of achieving may represent one of the psychological factors associated with the link between positive attitudes toward breastfeeding and breastfeeding intentions. Encouraging greater appreciation of body functionality during the breastfeeding experience may represent a potential direction for future research and intervention development.

From a theoretical and practical perspective, these findings may offer preliminary directions for future research. In particular, future studies could investigate whether higher levels of positive body image, and specifically functionality appreciation, are associated with breastfeeding intentions and whether such intentions translate into actual breastfeeding behaviors over time. These questions would benefit from longitudinal and experimental designs. In this context, the development and evaluation of interventions targeting functionality appreciation may represent a promising avenue for future research, while remaining cautious about causal interpretations.

## 5. Strengths, Limitations, and Future Directions

The present study has several strengths that should be acknowledged. First, it addresses an underexplored area by examining the role of functionality appreciation in the context of breastfeeding intentions during the postpartum period. Second, it integrates constructs from positive body image and breastfeeding research, offering a novel perspective on their interrelations.

This study was not without limitations. First, the use of a cross-sectional design prevents any inference regarding causality or temporal relationships between the variables. Accordingly, the mediation results should be interpreted as statistical and not as evidence of temporal or causal mechanisms. For instance, although we found that attitudes toward breastfeeding were related to higher functionality appreciation levels, we cannot determine whether breastfeeding attitudes led to greater functionality appreciation or whether women with higher functionality appreciation were more likely to report stronger breastfeeding intentions over time. Furthermore, the present study focused on breastfeeding intention rather than actual breastfeeding behavior, and, therefore, conclusions regarding behavioral outcomes should be drawn with caution.

Moreover, the use of a convenience sampling strategy may limit the generalizability of the findings. Participants were recruited online and were predominantly Italian, highly educated, and mostly married or cohabiting, which may reflect a relatively homogeneous and socioeconomically advantaged group. This may have introduced self-selection bias and may limit the applicability of the findings to more diverse populations. Given that breastfeeding behaviors are known to be influenced by socioeconomic and cultural factors, caution is warranted when generalizing these results to populations with different demographic and contextual characteristics. Future studies should aim to recruit more diverse and representative samples to enhance the external validity of the findings.

Women were also assessed at different points in the postpartum period (within 1–3 months of birth), so their body image, body size, and breastfeeding experiences may have been inherently different. Assessing women at the same time postpartum may help address this limitation.

Furthermore, the operationalization of previous breastfeeding experience through the multiplication of frequency and perceived quality represents an exploratory approach. While this choice was intended to capture both behavioral and experiential aspects of breastfeeding, its interpretation is not entirely straightforward. Moreover, given the predominance of primiparous women in the sample, this variable may have limited variability and applicability. Therefore, findings involving this variable should be interpreted with caution.

Moreover, although several sociodemographic and obstetric variables were assessed (e.g., BMI, parity, type of delivery), only previous breastfeeding experience was included as a covariate in the model, which may limit the comprehensiveness of the analyses.

Additionally, it is also possible that other factors not directly measured in the present study may play a role in shaping the link between attitudes and intentions. These may include social, personal, and structural variables (e.g., breastfeeding self-efficacy, social support, return to work, workplace conditions, and access to breastfeeding support services) that could influence how positive attitudes are translated into behavioral intentions. Future research should include a broader range of sociodemographic, psychosocial, and contextual variables to provide a more comprehensive understanding of breastfeeding behaviors.

Overall, these limitations highlight the need for cautious interpretation of the findings and for future studies adopting longitudinal and more diverse designs.

## 6. Conclusions

This study highlights the associations between breastfeeding attitudes, functionality appreciation, and breastfeeding intentions among postpartum women, also considering the role of previous breastfeeding experience. The findings underscore the relevance of considering positive body image and women’s appreciation of their bodies’ physiological capabilities in the context of breastfeeding.

Breastfeeding attitudes, body functionality appreciation, and breastfeeding intentions emerged as closely intertwined variables. Greater confidence in the body’s ability to nourish a child has been associated with stronger breastfeeding intentions. In line with this, the present findings indicate that these variables are meaningfully associated in postpartum women.

These insights may be useful for informing future research and for generating hypotheses regarding potential psychological factors associated with breastfeeding intentions. Further research is needed to examine the role of functionality appreciation in breastfeeding practices, particularly in relation to breastfeeding frequency and duration, using longitudinal and experimental designs.

In this context, interventions targeting favorable attitudes toward breastfeeding and greater awareness of body functions may represent a promising direction for future investigation. However, such implications should be interpreted with caution, given that the present study focused on breastfeeding intentions rather than actual behavior. Future studies are needed to determine whether these psychological factors are associated with breastfeeding behaviors over time and to clarify their potential role within broader clinical and public health approaches.

## Figures and Tables

**Table 1:** Sociodemographic and birth-related characteristics.

Variable		Mean ± SD or n (%)
Age		34.11 ± 2.19
Education level		
	Middle school	19 (6.1%)
	High school	110 (36.2%)
	Undergraduate education (Bachelor’s degree)	82 (27.1%)
	Postgraduate education (Master’s degree, post-lauream specialization courses, Ph.D.)	94 (30.8%)
Marital status		
	Unmarried	8 (2.6%)
	Married/cohabiting	266 (87.2%)
	Separated/divorced	31 (10.2%)
Nationality		
	Italian	299 (98%)
	Foreign	6 (2%)
Place of residence		
	Italy	305 (100%)
Parity		
	Primiparous	266 (74.3%)
	Multiparous	79 (25.7%)
Type of delivery		
	Spontaneous vaginal delivery	175 (57.4%)
	Induced vaginal delivery	68 (22.2%)
	Elective cesarean section	52 (16.9%)
	Emergency cesarean section	10 (3.3%)

**Table 2:** Descriptive statistics and correlations among the study variables.

	1.	2.	3.	4.	5.	M (SD)	Skewness (Kurtosis)
1. BMI	-					27.31 (3.61)	0.9 (1.5)
2. Breastfeeding attitudes	0.02	-				5.21 (1.12)	−0.8 (0.7)
3. Functionality appreciation	−0.08	0.51 ***	-			3.78 (0.74)	−0.6 (0.5)
4. Breastfeeding intentions	−0.06	0.63 ***	0.41 ***	-		5.48 (1.39)	−0.9 (0.4)
5. Previous breastfeeding experience	0.10	0.78 ***	0.66 ***	0.71 ***	-	4.81 (2.11)	0.7 (0.1)

*** *p* < 0.001. Note: Descriptive statistics refer to mean item scores for all multi-item scales.

## Data Availability

The data presented in this study are available from the corresponding author upon reasonable request due to the ongoing nature of the longitudinal study and related data management considerations.
